# Efficacy of pegaspargase, etoposide, methotrexate and dexamethasone in newly diagnosed advanced-stage extra-nodal natural killer/T-cell lymphoma with the analysis of the prognosis of whole blood EBV-DNA

**DOI:** 10.1038/bcj.2017.88

**Published:** 2017-09-15

**Authors:** J-H Liang, L Wang, R Peter Gale, W Wu, Y Xia, L Fan, J-Y Li, W Xu

**Affiliations:** 1 Department of Hematology, the First Affiliated Hospital of Nanjing Medical University, Jiangsu Province Hospital, Collaborative Innovation Center for Cancer Personalized Medicine, Nanjing, China; 2 Haematology Research Centre, Division of Experimental Medicine, Department of Medicine, Imperial College London, London, UK

Extra-nodal natural-killer (NK)/T-cell lymphoma (ENKTL) is an aggressive disease common in Asia but rare in the West. More than two-thirds of persons have stage-I/II disease of the upper aero-digestive tract.^1, 2, 3^ These persons often respond to radiation therapy with or without anti-cancer drugs but relapse is common.^4, 5^ Anthracycline-based regimens such as CHOP (cyclophosphamide, doxorubicin, vincristine and prednisone) are often used to treat advanced-stage disease but are ineffective with overall survival (OS) less than a year.^3^ Recently, L-asparaginase-based regimens were shown to be more active.^6, 7, 8^ The SMILE regimen (L-asparaginase, etoposide, methotrexate, ifosfamide and dexamethasone) regimen is reportedly effective in newly diagnosed persons with advanced-stage disease.^7, 8^ A regimen of modified SMILE was also reported to be effective by Yang *et al.*^6^ We tested a regimen of PEMD (pegaspargase, etoposide, methotrexate and dexamethasone) in 32 newly diagnosed advanced-stage subjects with ENKTL.

Thirty-two consecutive subjects with newly diagnosed advanced-stage (stages III–IV) ENKTL were prospectively studied from July 2010 to December 2015. The study was approved by the Ethics Committee of the First Affiliated Hospital of Nanjing Medical University. Subjects (32 patients) gave written informed consent in accordance with the Declaration of Helsinki. Biopsies were reviewed by ⩾2 experienced pathologists and diagnosis was based on WHO criteria.^1^ Baseline clinical variables including age, sex, Ann Arbor stage, serum lactate dehydrogenase, blood Epstein-Barr virus (EBV)-DNA levels (EBV-DNA copy number in whole blood was quantified by a real-time PCR based on the amplification of *EBNA1* gene with the cut-off value of 5000 copies/ml), B-symptoms, extra-nodal sites of disease, Eastern Cooperative Oncology Group (ECOG) performance score, bone marrow involvement, distant lymph-node involvement, International Prognostic Index (IPI) scores and prognostic index of natural killer lymphoma (PINK).^9^

pegaspargase, etoposide, methotrexate and dexamethasone was given as follows: methotrexate, 3.0 g/m^2^ i.v. over 6 h on day 1; etoposide, 100 mg/m^2^ i.v. on days 2–4; dexamethasone, 40 mg i.v. on days 1–4; pegaspargase, 2500 U/m^2^ i.m. on day 2. Subjects received 4–6 cycles of PEMD every 3 weeks. Patients could receive autologous or allogeneic hematopoietic stem cell transplantation after achieving complete remission or partial remission. The decision was made according to the discretion of the treating physician, mainly on the basis of the patient’s age, comorbidities, economy and wishes.

Response criteria are reported using standard criteria.^10^ Subjects were evaluated after three cycles of PEMD, after completing PEMD and every 3 months for 2 years thereafter. Responses were classified as complete response (CR), unconfirmed CR (CRu), partial response (PR), stable disease (SD) and progressive disease (PD). Physical exam and laboratory tests were used to evaluate adverse reactions and toxicities. Toxicities were graded according to the National Cancer Institute Common Toxicity Criteria, version 3.0.

Primary co-endpoints were response and survival. Overall response rate (ORR) was defined as the rate of CR/CRu and PR. Progression-free survival (PFS) was defined as the interval from study-entry to first progression. OS was defined as interval from study-entry to death from any cause. Secondary endpoints were proportion of subjects completing planned therapy (4–6 cycles of PEMD regimen) and frequencies of adverse events. Follow-up was through August 2016. Kaplan–Meier method was used to calculate PFS and OS. The log-rank test was used to analyze survival differences between the cohorts. Statistical analyses were performed using MedCalc for Windows, version 12.0.4.0 (MedCalc Software, Mariakerke, Belgium). *P*-values <0.05 were considered significant.

Baseline variables are indicated in Table 1. Median age was 48 years (range, 17–73 years). There were 25 males (78%). Nine subjects (28%) had non-nasal NK/T-cell lymphoma including five skin involvement, one testis involvement, one muscle involvement and two gastrointestinal tract involvement. Fourteen subjects (44%) had elevated serum lactate dehydrogenase levels. Thirteen subjects (41%) had higher level of EBV-DNA in whole blood (>5000 copies/ml). Ten patients (31%) had distant lymph-node involvement. Twenty-three subjects (72%) had B-symptoms and 17 (53%) had an IPI score ⩾3. Nineteen patients (59%) had a PINK score ⩾2.

Subjects received a median of four courses of PEMD (range, 1–6). Median follow-up is 48 months (range, 13–74 months). Only four patients had autologous HSCT. Three patients (two with non-nasal type received only one cycle and one with nasal type received two cycles) had early death (within 3 months after the diagnosis) due to haemo-phagocytic lympho-histiocytosis. Five patients (four with nasal type and one with non-nasal type) had disease progression after three cycles of PEMD. In summary, ORR was 75% (95% CI 57–89%) in the 32 subjects with advanced-stage disease including CR/CRu in 15 (47% (95% CI 29–65%)) and a PR in 9 (28% (14–47%)). For all the 32 patients, 4-year PFS was 44% (95%CI 25–63%) and OS 51% (95% CI 32–70%).

Table 2 lists grade-3/4 adverse events. Hematologic adverse events occurred in all subjects. Grade-1/2 hypo-fibrinogenemia was observed in 90% of subjects. Hematologic grade-3 toxicities included leukopenia (*N*=8), neutropenia (*N*=8), anemia (*N*=2) and thrombocytopenia (*N*=2). The most common non-hematologic grade-3 toxicities were hypo-fibrinogenemia (*N*=3) and infection (*N*=3). Hematologic grade-4 toxicities included leukopenia (*N*=3), neutropenia (*N*=3), anemia (*N*=1) and thrombocytopenia (*N*=1). Non-hematologic grade-4 toxicities included hypo-fibrinogenemia (*N*=1), infection (*N*=2) and hyper-bilirubinemia (*N*=1). There were no treatment-related deaths.

In order to select prognostic variables for advanced-stage ENKTL patients who were given PEMD regimen, univariate and multivariate cox regression analysis were conducted for both PFS and OS. By univariable analysis, factors predictive of both OS and PFS included PINK score above 1, IPI score above 2, elevated lactate dehydrogenase level and EBV-DNA-positivity. Factors significantly associated with PFS and OS in univariate analysis were entered into multivariate analysis. EBV-DNA-positivity and PINK score above 1 remained significant for PFS (HR, 5.19, 95% CI: 1.73–15.52, *P*<0.01 and HR, 5.60, 95% CI: 1.46–21.54, *P*=0.01) and OS (HR, 6.97, 95% CI: 2.05–23.63, *P*<0.01and HR, 4.56, 95% CI: 1.12–18.50, *P*=0.03) while IPI score above 2 only had significant trend for OS (HR, 5.02, 95% CI: 0.95–26.5, *P*=0.06), not for PFS (HR, 1.77, 95% CI: 0.48–6.54, *P*=0.39) (Table 3).

Among all the 32 patients, four patients (patients 1–4) experienced early death. Therefore, no dynamic quantitative changes in EBV-DNA with therapy were observed for these four patients. The dynamic continuous quantitative changes of all the other 10 pretreatment EBV-DNA-positive and all 18 EBV-DNA-negative patients with therapy, which were classified as CR/CRu, PR and no response (NR, included SD and PD), were shown in Figure 1. The three pretreatment EBV-DNA-positive patients (patients 4–6) who experienced PD at the mid-therapy had re-elevated EBV-DNA levels (Figure 1a). Among the seven pretreatment EBV-DNA-positive patients who achieved more than PR at the end of the therapy, three patients who experienced PD after the completion of the PEMD regimen also had re-elevated EBV-DNA levels (Figure 1b). One patient (patient 16) had re-elevated EBV-DNA levels among the two EBV-DNA-negative patients (patients 15 and 16) who had PD at the mid-therapy (Figure 1c). Among the 16 EBV-DNA-negative patients who achieved more than PR at the end of the therapy, two patients (patients 20 and 21) who experienced PD after the completion of the PEMD regimen also had re-elevated EBV-DNA levels (Figure 1d).

There is no standard therapy for ENKTL. Most data are from retrospective analyses and small prospective series. Anthracycline-containing regimens have response rates of 40–60% with high subsequent failure rates.^11^ SMILE is the most studied protocol for advanced disease. In a phase-2 study in 20 subjects with advanced disease ORR was 80% (95% CI 56–94%) and 2-year PFS and OS about 45%.^7^ However, the significant toxicities that grade-4 neutropenia were occurred in 92% patients and serious infections were occurred in 31% patients limited the application of this regimen in those not well-fit patients.

In our study, the 32 subjects with advanced disease had an ORR rate of (75% (95% CI 57–89%)) which was similar to that of SMILE.^7, 12^ Four-year PFS was 44% (95% CI 25–63%) and OS 51% (95% CI 32–70%), results similar to SMILE.^7, 11^ PEMD appeared better tolerated than SMILE with 57% (44, 70%) grade-3/-4 neutropenia (*P*<0.001) and 21% (11, 33%) grade-3/-4 infection (*P*<0.001). The decreased toxicity of PEMD likely results from deleting ifosfamide and using pegaspargase instead of L-asparaginase.

Circulating EBV-DNA in blood is derived from necrotic or apoptotic tumor cells, and thus viral DNA in the whole blood or plasma is strongly associated with survivals and treatment outcomes in patients with ENKTL which have been confirmed in many studies.^9, 13, 14^ Similar results were also observed in the present study specially for advanced ENKTL patients who were treated with PEMD regimens. Furthermore, the amount of circulating viral DNA might show the burden and replication of tumors, and it might be undetectable in patients with small tumor burdens or those in whom proliferation is less active. Therefore, the dynamic quantitative changes in EBV-DNA with therapy were also observed in the present study. Patients who experienced PD always had re-elevated EBV-DNA levels in the blood. Therefore, circulating EBV-DNA level was an important prognostic and monitoring tumor markers, which has been added in the prognostic index for natural killer lymphoma (PINK-E) reported by Kim *et al.*^9^

In conclusion, PEMD is safe and effective in persons with newly diagnosed advanced-stage ENKTL, especially for those not well-fit patients. Circulating EBV-DNA levels is an important prognostic and monitoring marker for advanced-stage ENKTL patients who treated with PEMD regimen. Larger prospective studies are needed to more precisely define toxicities and estimate efficacy and to compare PEMD with other regimens.

## Figures and Tables

**Figure 1:**
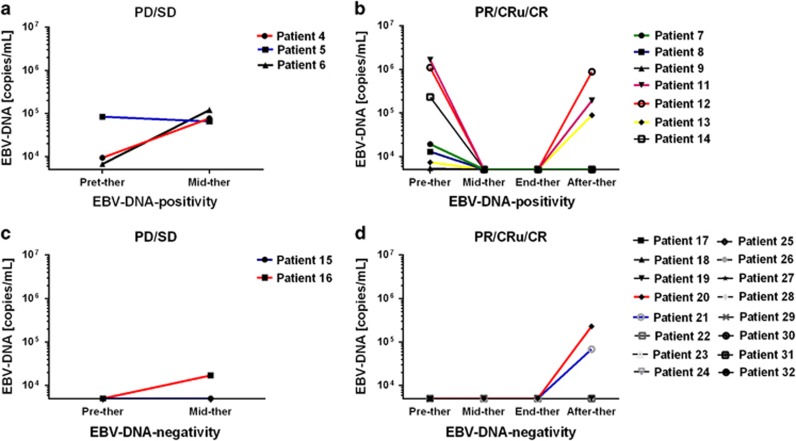
Serial analysis of quantitative Epstein–Barr virus (EBV)-DNA in 28 patients categorized into two groups: stable disease (SD)/progression disease (PD) (**a**, **c**), and complete response (CR)/CR undefined (CRu)/partical response (PR) (**b**, **d**).

**Table 1:** The baseline characteristics of 32 patients

	*N*	*%*
Age Median (Range)	48 (17–73) years	
Male	25	78
Nasal subtype	23	72
Age >60 years	9	28
Elevated serum LDH	14	44
ECOG score ⩾2	7	22
Distant lymph-node involvement	10	31
IPI score ⩾3	17	53
PINK ⩾2	19	59
B-symptoms	23	72
EBV-DNA >5000 copies/ml	13	41

Abbreviations: EBV, Epstein-Barr virus; ECOG, Eastern Cooperative Oncology Group; IPI, International Prognostic Index; LDH, lactate dehydrogenase; PINK, prognostic index of natural killer lymphoma.

**Table 2:** Adverse events grade-3/-4 (*N*=32)

	*Grade-3*	*Grade-4*	*Total*
*Hematologic*			
Leukopenia	8 (25%)	3 (9%)	11 (34%)
Neutropenia	8 (25%)	3 (9%)	11 (34%)
Anemia	2 (6%)	1 (3%)	3 (9%)
Thrombocytopenia	2 (6%)	1 (3%)	3 (9%)
			
*Non-hematologic*			
Hypo-fibrinogenemia	3 (9%)	1 (3%)	4 (13%)
Hypo-albuminemia	2 (6%)	0 (0%)	2 (6%)
Hyper-bilirubinemia	2 (6%)	1 (3%)	3 (9%)
Aspartate transaminase increase	0 (0%)	0 (0%)	0 (0%)
Alanine transaminase increase	0 (0%)	0 (0%)	0 (0%)
Infection	3 (9%)	2 (6%)	5 (15%)
Appetite loss	1(3%)	0 (0%)	1 (3%)
Nausea	0 (0%)	0 (0%)	0 (0%)
Diarrhea	0 (0%)	0 (0%)	0 (0%)
Vomiting	0 (0%)	0 (0%)	0 (0%)

**Table 3:** Cox regression analysis for PFS and OS for advanced-stage ENKTL patients treated with PEMD (*N*=32)

*Characteristic*	*PFS*				*OS*			
	*Univariate analysis*		*Multivariate analysis*		*Univariate analysis*		*Multivariate analysis*	
	*HR (95% CI)*	P	*HR (95% CI)*	P	*HR (95% CI)*	P	*HR (95% CI)*	P
Male	2.30 (0.52–10.01)	0.27	—	—	1.93 (0.43–8.63)	0.39	—	—
Nasal subtype	1.45 (0.50–4.20)	0.49	—	—	1.67 (0.56–4.99)	0.36	—	—
Age >60 years	2.15 (0.77–5.96)	0.14	—	—	2.67 (0.92–7.80)	0.07	—	—
LDH >ULN	3.40 (1.17–9.88)	**0.02**	2.01 (0.60–6.74)	0.26	3.66 (1.13–11.75)	**0.03**	1.80 (0.51–6.36)	0.36
ECOG score ⩾2	0.59 (0.17–2.07)	0.41	—	—	0.81 (0.22–2.92)	0.74	—	—
Distant lymph-node involvement	1.11 (0.37–3.32)	0.85	—	—	0.81 (0.27–2.49)	0.72	—	—
IPI score ⩾3	3.67 (1.17–11.47)	**0.03**	1.77 (0.48–6.54)	0.39	7.84 (1.73–35.49)	**<0.01**	5.02 (0.95–26.5)	**0.06**
PINK ⩾2	4.03 (1.46–11.08)	**0.02**	5.60 (1.46–21.54)	**0.01**	3.46 (1.21–9.88)	**0.04**	4.56 (1.12–18.50)	**0.03**
B-symptoms	1.74 (0.49–6.13)	0.39	—	—	2.33 (0.52–10.43)	0.27	—	—
EBV-DNA-positivity	3.32 (1.11–9.93)	**0.02**	5.19 (1.73–15.52)	**<0.01**	3.88 (1.26–11.95)	**0.01**	6.97 (2.05–23.62)	**<0.01**
Extranodal site >1	1.58 (0.59–4.25)	0.37	—	—	2.28 (0.76–6.83)	0.14	—	—

Abbreviations: CI, confidence interval; EBV, Epstein-Barr virus; ECOG, Eastern Cooperative Oncology Group; ENKTL, extra-nodal natural-killer (NK)/T-cell lymphoma; HR, hazard ratio; IPI, International Prognostic Index; LDH, lactate dehydrogenase; OS, overall survival; PEMD, pegaspargase, etoposide, methotrexate and dexamethasone; PFS, progression-free survival; PINK, prognostic index of natural killer lymphoma; ULN, upper limit of normal. Bold signifies *P*<0.05.

## References

[bib1] Swerdlow SH, Campo E, Harris NL, Jaffe ES, Pileri SA, Stein H et al. World Health Organization classification of tumours of haematopoietic and lymphoid tissue. IARC Press: Lyon, 2008.

[bib2] Au WY, Weisenburger DD, Intragumtornchai T, Nakamura S, Kim WS, Sng I et al. Clinical differences between nasal and extranasal natural killer/T-cell lymphoma: a study of 136 cases from the International Peripheral T-cell Lymphoma Project. Blood 2009; 113: 3931–3937. 19029440 10.1182/blood-2008-10-185256

[bib3] Suzuki R, Suzumiya J, Yamaguchi M, Nakamura S, Kameoka J, Kojima H et al. Prognostic factors for mature natural killer (NK) cell neoplasms: aggressive NK cell leukemia and extranodal NK cell lymphoma, nasal type. Ann Oncol 2010; 21: 1032–1040. 19850638 10.1093/annonc/mdp418

[bib4] Li YX, Yao B, Jin J, Wang WH, Liu YP, Song YW et al. Radiotherapy as primary treatment for stage 1E and IIE nasal natural killer/T cell lymphoma. J Clin Oncol 2006; 24: 181–189. 16382127 10.1200/JCO.2005.03.2573

[bib5] Kim SJ, Kim K, Kim BS, Kim CY, Suh C, Huh J et al. Phase II trial of concurrent radiation and weekly cisplatin followed by VIPD chemotherapy in newly diagnosed, stage IE to IIE, nasal, extranodal NK/T-cell lymphoma: Consortium for Improving Survival of Lymphoma study. J Clin Oncol 2009; 27: 6027–6032. 19884539 10.1200/JCO.2009.23.8592

[bib6] Yang L, Liu H, Xu XH, Wang XF, Huang HM, Shi WY et al. Retrospective study of modified SMILE chemotherapy for advanced-stage, relapsed, or refractory extranodal natural killer (NK)/T cell lymphoma, nasal type. Med Oncol 2013; 30: 720. 24062259 10.1007/s12032-013-0720-7

[bib7] Yamaguchi M, Kwong YL, Kim WS, Maeda Y, Hashimoto C, Suh C et al. Phase II study of SMILE chemotherapy for newly diagnosed stage IV, relapsed, or refractory extranodal natural killer (NK)/T-cell lymphoma, nasal type: the NK-Cell Tumor Study Group Study. J Clin Oncol 2011; 29: 4410–4416. 21990393 10.1200/JCO.2011.35.6287

[bib8] Yamaguchi M, Suzuki R, Kwong YL, Kim WS, Hasegawa Y, Izutsu K et al. Phase I study of dexamethasone, methotrexate, ifosfamide, L-asparaginase, and etoposide (SMILE) chemotherapy for advanced-stage, relapsed or refractory extranodal natural killer (NK)/T-cell lymphoma and leukemia. Cancer Sci 2008; 99: 1016–1020. 18294294 10.1111/j.1349-7006.2008.00768.x PMC11158592

[bib9] Kim SJ, Yoon DH, Jaccard A, Chng WJ, Lim ST, Hong H et al. A prognostic index for natural killer cell lymphoma after non-anthracycline-based treatment: a multicenter retrospective analysis. Lancet Oncol 2016; 17: 389–400. 26873565 10.1016/S1470-2045(15)00533-1

[bib10] Kwong YL. Natural killer-cell malignancies: diagnosis and treatment. Leukemia 2005; 19: 2186–2194. 16179910 10.1038/sj.leu.2403955

[bib11] Chim CS, Ma SY, Au WY, Choy C, Lie AK, Liang R et al. Primary nasal natural killer cell lymphoma: long-term treatment outcome and relationship with the International Prognostic Index. Blood 2004; 103: 216–221. 12933580 10.1182/blood-2003-05-1401

[bib12] Kwong YL, Kim WS, Lim ST, Kim SJ, Tang T, Tse E et al. SMILE for natural killer/T-cell lymphoma: analysis of safety and efficacy from the Asia Lymphoma Study Group. Blood 2012; 120: 2973–2980. 22919026 10.1182/blood-2012-05-431460

[bib13] Kim HS, Kim KH, Kim KH, Chang MH, Ji SH, Lim DH et al. Whole blood Epstien–Barr virus DNA load as a diagnostic and prognostic surrogate: extranodal natural killer/T-cell lymphoma. Leuk Lymphoma 2009; 50: 757–763. 19330658 10.1080/10428190902803669

[bib14] Ito Y, Kimura H, Maeda Y, Hashimoto C, Ishida F, Izutsu K et al. Pretreatment EBV-DNA copy number is predictive of response and toxicities to SMILE chemotherapy for extranodal NK/T-cell lymphoma, nasal type. Clin Cancer Res 2012; 18: 4183–4190. 22675173 10.1158/1078-0432.CCR-12-1064

